# Therapeutic drug monitoring in clozapine treatment

**DOI:** 10.1192/j.eurpsy.2025.228

**Published:** 2025-08-26

**Authors:** P. Mohr

**Affiliations:** 1 National Institute of Mental Health - CZ, Klecany; 2 3rd Faculty of Medicine, Charles University, Prague, Czech Republic

## Abstract

**Abstract:**

Although still somewhat reluctantly used (and thus underutilized) by many clinicians, it is the well-evidenced fact that clozapine is an effective drug for treatment-resistant schizophrenia. In order to optimize its therapeutic potential and to minimize side effects, therapeutic drug monitoring (TDM) of plasma clozapine and N-desmethylclozapine is an essential clinical tool. TDM can also help to reveal insufficient adherence or individual differences in the rates of metabolism. Moreover, clozapine plasma levels can be affected by other factor, such as smoking or concomitant medication (fluvoxamine). In our study sample, we were able to demonstrate the relationship between the dosage and clozapine plasma levels and their impact on clinical efficacy and safety.

**Disclosure of Interest:**

None Declared

